# Time-to-Progression of NSCLC from Early to Advanced Stages: An Analysis of data from SEER Registry and a Single Institute

**DOI:** 10.1038/srep28477

**Published:** 2016-06-27

**Authors:** Ping Yuan, Jin Lin Cao, Azmat Rustam, Chong Zhang, Xiao Shuai Yuan, Fei Chao Bao, Wang Lv, Jian Hu

**Affiliations:** 1 Department of Thoracic surgery, The First Affiliated Hospital, School of Medicine, Zhejiang University, Hangzhou, 310003, China

## Abstract

The average time required for cancers to progress through stages can be reflected in the average age of the patients diagnosed at each stage of disease. To estimate the time it takes for non-small-cell lung cancer (NSCLC) to progress through different tumor, node and metastasis (TNM) stages and sizes, we compared the mean adjusted age of 45904 NSCLC patients with different stages and tumor sizes from Surveillance, Epidemiology and End Results (SEER) cancer registry database and our institute. Multiple-linear-regression models for age were generated adjusting for various factors. Caucasian, African-American and Asian patients with stage IA cancers were on average 0.8, 1.0 and 1.38 adjusted years younger, respectively, than those with stage IIIB cancers (p < 0.001). And these with T1a cancers were on average 0.84, 0.92 and 1.21 adjusted years younger, respectively, than patients with T3 cancers (p < 0.001). Patients with tumors measuring larger than 8 cm in diameter were on average 0.85 adjusted years older than these with tumors smaller than 1 cm (p < 0.001), with Caucasian demonstrating the shortest age span (0.79 years, P < 0.001). In conclusion, the time-to-progression of NSCLC from early to advanced stages varied among ethnicities, Caucasian patients demonstrating a more rapid progression nature of tumor than their African-American and Asian counterparts.

Lung cancer is the leading cause of cancer-related death in both men and women worldwide^1^. Most lung cancers (85%) are classified as non-small cell lung cancer (NSCLC), with adenocarcinoma and squamous cell carcinoma being the most common subtypes. The advancements in thoracic imaging have increased the incidence of detection of small solitary pulmonary nodules (SPNs)^2^,^3^. Treatment decisions for these lesions must weigh the risks and benefits of prompt identification of malignant nodules to avoid surgery in patients with benign nodules. Highlighting optimum management of SPN.

Given that a lesion’s growth pattern can largely reflect its malignancy, a better understanding of the natural development processes of the early clinical stages of lung cancer has instructive significance in present surveillance guidance for lung cancer screening when a small sized SPN is detected^4^. The growth nature of lung tumor has been examined in several studies by estimating the average volume doubling time (VDT)^5^,^6^,^7^,^8^. The overestimation of a solid nodules smaller than 6 mm by VDT indicates a degree of imprecision, and may yield uncertainty in doubling times that make benign and malignant nodules indistinguishable. Other studies have attempted to estimate tumor growth using experimental models. However, it is unclear that whether these data from the models can be accurately extrapolated to represent the growth properties of primary human lung cancers^5^,^7^.

If the average time required for NSCLC to progress through its different stages is long enough, the average age differences of the patients diagnosed at each stage can be presumed to be the stage-to-stage time intervals^9^. In our study, the data from the National Cancer Institute’s (NCI’s) Surveillance, Epidemiology and End Results (SEER) database and our institute were analyzed to estimate the stage-to-stage age intervals throughout NSCLC. The average adjusted age of patients with small and early stage lung cancers versus those with larger and advanced stage lung cancers were compared to estimate the average time it takes lung cancer to progress through its clinical stages.

## Patients and Methods

### Data source

The SEER database was queried for this study. SEER has been continuously collecting data since 1973 from 18 different registries that represent ∼28% of the United States population. Inclusion criteria for our study included patients aged >30 years, diagnosis of lung adenocarcinoma and squamous cell carcinoma based on pathologic confirmation. The searching codes are demonstrated in Supplementary Table 1. Following exclusion of patients with limited information, A total of 44,824 NSCLC patients with modified American Joint Committee on Cancer (AJCC) stages I-IV status (7^th^ edition^10^, depicted in Supplementary Table 2), diagnosed between 2004 and 2012, were identified in the SEER database. Demographic data collected included patients age at diagnosis (AAD), gender and race. Pathologic characteristics collected include primary tumor site, histology type, tumor size and neoplastic grade. Patient ethnicity was grouped by geographic distribution into Caucasian, African-American and Asian.

In addition, a cohort of 1,080 Chinese NSCLC patients who received surgical treatment between 2010 and 2012 in our department with the same criteria as the eligible patients in the SEER dataset were also included in this analysis. Ethical approval was obtained from the institutional review board of the First Affiliated Hospital of Zhejiang University. The need for informed consent from patients was waived due to the study’s retrospective nature.

### Statistics analysis

The primary goal of the present study was to identify factors associated with AAD and to estimate the average time-to-progression of lung cancer through its clinical stages using multiple linear regression models. Student’s t-tests were used to examine the patient and tumor characteristics. Differences in age by patient groups defined by tumor, node and metastasis (TNM) stage and tumor size were estimated with multivariable linear regression models that adjusted for sex, ethnicity, tumor location, histologic type and grade. Given that the quantitation of primary tumor burden is more accurate than the extent of metastatic disease. This analysis only focused on estimating the average time-to-progression of NSCLC among patients who had localized or locally advanced disease (stage I~III lung cancer). All of the analyses were completed using SPSS (Version 22.0).

## Results

### Patient characteristics

The basic information of patients in the SEER database and our institute is shown in Tables 1 and 2. Caucasians were significantly older at the time of diagnosis than African-Americans (mean difference (MD), years), 95%CI, (3.37, 3.06~3.68) and Asians (3.34, 2.99~3.69) (p < 0.001 for both). Males with NSCLC were 0.47 years older than females (MD, 95%CI: 0.28~0.66, p < 0.001). Patients with undifferentiated tumors (68.15, 67.2~69.1) were, on average, 0.84 adjusted years younger (95%CI: −0.10~1.78, p = 0.08) than those with higher neoplastic grade (well, moderately and poorly). Those patients whose tumors located in the main bronchus is younger compared to other patients. (P < 0.001 for all).

The relationship between patients’ race and tumor characteristics was showed in Tables 3 and 4. The results are similar to overall analysis, but significant difference in AAD between male and female was not found in Caucasians (Table 3). We compared the tumor size by patient demographics (Table 4). African-Americans had larger tumors on average than Caucasians (mean tumor diameter, 38.89 mm (38.25~39.52) vs. 35.78 mm (35.56~36.00) and Asians (33.46 mm (32.82~34.10), p < 0.001 for both). Males had larger primary tumors on average than females (38.63 mm (38.35~38.92) vs. 33.13 mm (32.86~33.39) p < 0.001). Tumor of squamous cell carcinoma is larger than tumor of adenocarcinoma (42.47 mm (42.12~42.82) vs 32.41 mm (32.19~32.64), p < 0.001). Patients with well-differentiated (25.71 mm (25.33~26.09)) and moderate differentiated cancers (33.86 mm (33.57~34.15)) had smaller tumors on average than patients with poorly differentiated tumors (41.73 mm (41.41~42.04); p < 0.001 for both). No significant difference in size was found between poorly differentiated tumors and undifferentiated (42.70 mm (40.60~44.80)) tumors. The average size of tumors located in the main bronchus (49.49 mm (48.22~50.75)) was significantly larger than those in lobe (p < 0.001 for all comparisons, Table 3). Tumors located in the middle lobe (31.87 mm (31.09~32.64)) was smaller than those in upper (35.73 mm (35.48~35.98)) and lower lobe (36.00 mm (35.66~36.35, p < 0.001 for both). No significant difference in size was found between tumors in upper and lower lobes.

We compared the tumor characteristics between Asian patients in SEER database and our institute (Supplementary Table 3). The patients in our institute is younger compared to the patients in the SEER database (60.71 years vs. 68.60 years), and had more moderately-differentiated tumor (74.7% vs. 41.9%), more squamous cell carcinoma (25.8% vs. 20%, p < 0.001). In SEER database, the majority of Asian group includes Japanese, Korean and Vietnamese. Whereas all the patients in our institute is Chinese patients, the different nation in the two cohorts might explain the difference. All this factors has been adjusted in the linear regression model.

### Relationships between patient age and overall, T and N stages

To estimate the time it takes for an early stage cancer to progress to advanced stage cancers, we compared the average adjusted age of patients with localized or locally advanced cancers without distant metastases after adjusting for patients ethnicity, sex, tumor location and grade (Table 5). Patients with stage IA cancers (age: 68.96 adjusted mean years) were on average 0.83 adjusted years younger (p < 0.001) than patients with IIIB cancers (age: 69.79 adjusted mean years). This difference was more evident when comparing African-American and Asian patients, the average adjusted AAD of these patients with IIIB disease were 1.00 and 1.38 years older, respectively, than patients with IA disease (p < 0.001 for all).

Interestingly, among all the patients, the age/stage relationship was nonsignificant for patients with stage IV cancers (distant metastases cancers) versus stage IA cancers (Supplementary Table 4). Stratification analyses by ethnicity found no significant age/stage relationship in Caucasian and African-American, Only Asian patients with stage IV cancers were older than their counterparts with stage IA cancers (0.79 years, p < 0.001). The lack of age difference between stage IV patients and stage IA patients may largely due to the fact that the primary tumor size in patients with localized or locally advanced lung cancer can better demonstrate the overall tumor burden and disease duration than it is in patients with metastatic lung cancer. Besides, the tumor size of metastatic lung tumor is mainly measured by imaging tests. The accuracy of imaging tests of lesion size in patients with metastatic lung cancer is inferior to pathological measurements of the primary cancer performed in patients who received surgical treatment.

We therefore explored the differences in the adjusted age of patients with localized or locally advanced disease according to their primary tumor T stage (Table 5). Among patients diagnosed with stage IA to IIIB cancers, the average adjusted age of patients with T1b, T2a, T2b, T3 and T4 tumors was significantly older than that of patients with T1a tumors (by 0.08, 0.15, 0.63, 0.62 and 0.92 years, respectively, p < 0.001 for all). Interestingly, the age span from low-to-high T stage did not demonstrate an increasing trend in a general term; specifically, the age differences became less evident when T3 were compared with the T1a stage in all ethnics. The adjusted age of Caucasian, African-American and Asian patients with T3 tumors was significantly younger than that of patients with T2b tumors (by 0.17, 0.51 and 0.19 years, p < 0.001 for all). This decreased age difference among patients with T3 lung cancers indicates that a tumor’s invasiveness may not be time dependent. Small aggressive tumors may also occur in young patients without a time course of invasion. Similar to overall stage comparisons, the age difference for the T/age comparison showed that African-Americans and Asians demonstrated longer age spans (from T1a~T3) than that of Caucasians; specifically, 0.92, 1.21 and 0.84 years (p < 0.001 for all), respectively. Those results indicate that lung cancer develops more rapidly in Caucasians than in African-Americans and Asians. Subgroup analysis of Asian patients in SEER database and patients in our institute showed a similar result (Supplementary Table 5).

We further explored the age/T stage relationship within the specific and throughout TNM stages in all patients (Supplementary Tables 6 and 7). The age difference between the T stages within the same and throughout TNM stages showed similarly results. However, due to limited T1a patients in late TNM stages, the Asian patients failed to demonstrated significant age difference in the same TNM stages.

We analyzed the time required for lung cancer progression with different histologic types. The results showed that it takes less time for early stage lung adenocarcinoma to progress to advanced stage. In patients with adenocarcinoma, the average age of stage IIIB patients is 1.17 years younger (p < 0.001) than patients with IA disease, whereas in squamous cell carcinoma patients, the time span is 2.5 years (p < 0.001) (Table 6). The age difference for the T/age comparison showed that squamous cell carcinoma patients demonstrated longer age spans (from T1a~T3) than that of adenocarcinoma; specifically, 1.8 and 0.92 years (p < 0.001 for both).

Some expected patterns in the patient age/N stage relationship were also revealed. Among patients diagnosed with stage IA to IIIB cancers, the average adjusted age of patients with N1, N2 and N3 tumors was significantly older than that of patients with N0 tumors (by 0.09, 0.12 and 0.56 years, respectively, p < 0.01 for all, Table 5). This difference was more evident in stratified analysis in African-American and Asian patients. African-American and Asian patients with N0 cancers were on average 0.77 and 0.93 adjusted years younger, respectively, than patients with N3 cancers (p < 0.001 for both).

### Relationships between patient age and tumor size

To exclude cofactors other than tumor size in T3 staging (Supplementary Table 2), we explored the relationships between patient age and primary tumor size alone to see if the similar results can be found. As we expected, similar trends were noted: Among patients with stage I, II, and III patients, there was a general trend within these stage subgroups for patients with larger tumors to be older than those with smaller tumors (Table 7). The mean adjusted age of patients with tumors measuring larger than 8 cm in diameter was on average 0.85 adjusted years older than those with tumors measuring ≤1 cm (p < 0.001). Whereas the age difference was not evident when comparing patients with tumors measuring 1 to 2 cm to that of smaller than 1 cm, even the tumors measuring 1 to 2 cm were on average 0.8 cm larger than tumors measuring ≤1 cm in size. A further stratification analysis by ethnicity showed similar trends and that Caucasians with tumor measuring >8 cm demonstrated the shortest age span (0.79 years younger, p < 0.001) to their counterparts with tumors measuring ≤1 cm and Asians demonstrated the longest (1.79 years younger, p < 0.001).

## Discussion

In the present study, the average AAD of lung cancer patients was compared after adjusting for cofactors to estimate the average time it takes lung cancers to grow within and through different stages and sizes. The small age difference between patients of early and advanced stage indicates that once a lung cancer lesion is detectable by tests, its growth and progression to more advanced stages of the disease is rapid, especially in Caucasians. We report a variation in lung cancer progression pattern for Caucasians, African-Americans and Asians, The relatively longer duration of growth in Asian patients is consistent with several studies from Asian institutes that have estimated the VDTs of lung cancer using imaging tests^6^,^8^,^11^,^12^. The heterogeneity of such patterns in other ethnicities should be validated through additional cohort studies.

Ethnic variations in lung cancer progression may be explained by many factors, including differences in smoking prevalence^13^, environmental exposures^14^, socioeconomic status^15^ and genetic backgrounds^16^. Regional differences in smoking prevalence may contribute to the disparities. It is reported that the mean of tumor VDT was significantly shorter in patients with a smoking history than in patients without a smoking history^17^. In addition to smoking status, ethnic variation of gene mutations may attribute to the discrepant progression patterns. Many biomarkers, including K-ras and epidermal growth factor receptor (EGFR) somatic mutations have clearly demonstrated different characteristics between NSCLC patients in Asian and Caucasian populations. K-ras mutation is predominantly observed in Caucasian patients^18^. The EGFR mutation rate is approximately 5~13% among Caucasians but 30~40% among East Asians.

The finding that the progression of lung cancer in Caucasians is far more rapid than in other ethnicities may explain why lung cancer mortality proved higher in developed countries such as Europe, North America, Australia and New Zealand, whereas Asia and Africa showed plateauing or decreasing rates^19^,^20^. Several studies have ascribed this mortality variation to the differences in the stage and degree of the tobacco epidemic^21^. Here, we presume that Caucasian patients might lose the best opportunities for surgery on their first visit due to the rapid progression feature.

The increase in age from low-to-high stages did not demonstrate a general trend. The exception of IIIA and IIIB versus IA stages could be linked to the similar results of specific T/age comparisons (T3/T4 (locally advanced tumors) versus the T1a stage). In addition, skip lymphatic metastasis (N2 of IIIA and IIIB stages) and small aggressive tumors (T3 of IIIA/IIIB stages) in young patients might also diminish the age differences between patients with stage I or II disease and stage III disease. Studies have reported that 5–25% of patients have N2 skip lymphatic metastasis^22^,^23^, 6–16% of small tumors (≤3 cm) in previous studies had lymphatic metastasis and 19.8~34.2% of small size tumors have proven to be locally invasive tumors (T3/T4)^22^,^24^,^25^,^26^. Our dataset showed that 23.89% (5626/23552) of patients with small tumors (≤3 cm) had lymphatic metastasis and 15.7% (3703/23552) were locally invasive (T3 and T4) tumors. 641 of the primary lung tumors (stage I~III, 34845 patients, 1.8%) measuring smaller than 7 mm, 67 of them (10.5%) had lymphatic metastasis, 119 (18.6%) were locally advanced lesions (T2a, T3, T4) (Table 8). The data indicate that invasiveness cannot be overlooked in small sized lung tumors, the possible age independent factors should be noted in relation to the small age difference in high T stage and overall stages versus lower stages.

Evidence that the progression of lung cancers through their clinical stages is not a long-term course calls for more effort in detecting lung cancer while it is still at an early stage, especially in Caucasians. The principal response to “how often, and how long to screen” for high-risk patients is whether the benefit seen in the NLST would be modified by screening for longer periods at different intervals than those used in the NLST. The current recommendations from US Preventive Services Task Force (USPSTF), which are mainly based on the NLST trial, has advocated that screening should be undertaken annually in a pre-specified groups of individuals. Duffy and colleagues estimated the likely effects of annual and biennial screening programs, suggesting that the benefit of biennial screening is subject to additional uncertainty but the issue merits further empirical research^27^. Although the 2-year probability of developing lung cancer was 0.4% in NELSON (The Dutch-Belgian Randomized Lung Cancer Screening Trial) participants with no pulmonary nodules detected, our analysis indicates that it take less than 1 year for a small tumor lesion in Caucasians to progress to advanced stage disease, suggesting a high risk of delayed diagnosis by annual screening of lung cancer in this population. Besides, the time to progression of NSCLC to advanced stage takes around 1 to 1.5 years for African-Americans and Asians, Thus further studies are needed to validate whether a screening interval of at least 2 years is safe to apply in these individuals. In addition, considering the growth nature of tumor in our dataset varied among ethnicities, potential benefit may lies in customized surveillance strategies for monitoring SPNs in different ethnicity.

Our study confirmed other prior associations between lung cancer risk factors and AAD. African-Americans were diagnosed with lung cancer at an older age than other ethnic groups, patients with squamous cell lung cancer to be older and had larger tumors than adenocarcinoma. We also found that larger primary lung cancers were more likely to be poorly differentiated and undifferentiated, consistent with the hypothesis that larger tumors are more vulnerable to tumor hypoxia, and in turn influence the differentiation state of the tumor^28^.

Strengths of this study include the breadth of the SEER database creates a more representative population of patients and greater generalizability of results, and the large sample size provided sufficient statistical power to thoroughly understand the natural history of progression of adenocarcinoma and squamous cell carcinoma of lung. A limitation is the fact that smoking history is not provided in the SEER data source, which would have helped understanding whether these ethnic differences can be accounted for by smoking status alone, or whether they imply more fundamental biological differences.

In conclusion, we demonstrated that lung cancer progression is rapid by comparing the adjusted average ages of patients with localized or locally advanced lung cancer at diagnosis. The Caucasian NSCLC patients showed a far more rapid progression than the African-American and Asian patients, calling for customized strategies of SPNs surveillance in lung cancer screening among different ethnic groups.

## Additional Information

**How to cite this article**: Yuan, P. *et al*. Time-to-Progression of NSCLC from Early to Advanced Stages: An Analysis of data from SEER Registry and a Single Institute. *Sci. Rep*. **6**, 28477; doi: 10.1038/srep28477 (2016).

## Supplementary Material

Supplementary Information

## Figures and Tables

**Table 1:** Characteristics of all Surveillance, Epidemiology and End Results(SEER) program and in-institute participants, by cancer stages.

	All patient N = 45904	Stage IA N = 12769	Stage IB N = 6658	Stage IIA N = 3384	Stage IIB N = 3194	StageIIIA N = 6911	Stage IIIB N = 1929	Stage IV n = 11059
Age at diagnosis—median(Range)	70(30,112)	70(30,112)	71(30,97)	69(30,96)	70(31,101)	69(30,96)	69(31,94)	69(30,102)
Age at diagnosis—mean(SD)	68.98(10.0)	69.08(10.0)	70.05(10.30)	68.79(10.43)	69.22(10.22)	68.74(10.41)	68.05(10.75)	68.52(10.98)
Race—Number(%)								
Caucasian	37542(81.8)	10634(83.3)	5464(82.1)	2743(81.1)	2695(84.4)	5568(80.6)	1553(80.5)	8875(80.3)
African-American	4745(10.3)	1054(8.3)	605(9.1)	338(10.0)	301(9.4)	768(11.1)	256(13.3)	1423(12.9)
Asian	3627(7.9)	1081(8.5)	589(8.8)	303(9.0)	198(6.2)	575(8.3)	120(6.2)	761(6.9)
Sex—Number(%)								
Male	23244(50.6)	5486(4.3)	3282(49.3)	1803(53.3)	1689(52.9)	3778(54.7)	1099(57.0)	6106(55.2)
Female	22660(49.4)	7283(5.7)	3376(50.7)	1581(46.7)	1505(47.1)	3132(45.3)	830(43.0)	4953(44.8)
Grade—Number(%)								
Well-differentiated	7456(16.2)	3971(31.1)	1165(17.5)	371(11.0)	525(16.4)	539(7.8)	97(5.0)	788(7.1)
Moderately-differentiated	18792(40.9)	5696(44.6)	3172(47.6)	1514(44.7)	1312(41.1)	2789(40.4)	692(35.9)	3617(32.7)
Poorly-differentiated	19183(41.8)	3041(23.8)	2270(34.1)	1466(43.3)	1322(41.4)	3503(50.7)	1118(58.0)	6463(58.4)
undifferentiated	473(1.1)	61(0.5)	51(0.8)	33(1.0)	35(1.1)	80(1.2)	22(1.1)	191(1.7)
Histology								
Squamous cell carcinoma	15989(34.8)	3266(25.6)	2309(34.7)	1413(41.8)	1358(42.5)	3023(43.7)	1011(52.4)	3608(32.6)
Adenocarcinoma	29915(65.2)	9497(74.4)	4349(65.3)	1971(58.2)	1835(57.5)	3888(56.3)	918(47.6)	7451(67.4)
Size(cm)—Number(%)								
size ≤ 1 cm	2429(5.3)	1672(13.1)	163(2.4)	61(1.8)	119(3.7)	175(2.5)	27(1.4)	211(1.9)
1 < size ≤ 2 cm	10987(23.9)	6665(52.2)	1063(16.0)	473(14)	583(18.3)	949(13.7)	156(8.1)	1098(9.9)
2 < size ≤ 3 cm	10136(22.1)	4431(34.7)	1238(18.6)	674(19.9)	455(14.2	1344(19.4)	237(12.3)	1757(15.9)
3 < size ≤ 5 cm	12160(26.5)	–	4194(63.0)	916(27.1)	590(18.5)	2204(31.9)	564(29.2)	3692(33.4)
5 < size ≤ 7	6446(14.0)	–	–	1260(37.2)	748(23.4)	1335(19.3)	549(28.5)	2554(23.1)
size>7 cm	3746(8.2)	–	–		699(219)	904(13.1)	396(20.5)	1747(15.8)
Site								
Upper lobe	68.79(10.3)	7691(60.2)	3970(59.6)	1857(54.9)	1955(61.2)	4280(61.9)	1258(65.2)	6705(60.6)
Middle lobe	68.36(10.8)	786(6.2)	412(6.2)	178(5.3)	143(4.5)	344(5.0)	88(4.6)	534(4.8)
Lower lobe	69.66(10.4)	4265(3.3)	2206(33.1)	1288(38.1)	1046(32.7)	2077(30.1)	454(23.5)	3343(30.2)
Main bronchus	65.82(11.2)	27(0.2)	70(1.1)	61(1.8)	50(1.6)	210(3.0)	129(6.7)	477(4.3)
T stage								
T1a	9970(21.7)	8310(65.1)	–	426(12.6)	–	546(7.9)	79(4.1)	545(4.9)
T1b	6317(13.8)	4459(34.9)	–	451(13.3)	–	593(8.6)	89(4.6)	725(6.6)
T2a	11983(26.1)	–	6658(100)	1230(36.3)	–	1711(24.8)	203(10.5)	2181(19.7)
T2b	3579(7.8)	–	–	1277(37.7)	392(12.3)	700(10.1)	129(6.7)	1081(9.8)
T3	7893(17.2)	–	–	–	2802(87.7)	1944(28.1)	238(12.3)	2910(26.3)
T4	6225(13.6)	–	–	–	–	1417(20.5)	1191(61.7)	3617(32.7)
N stage								
N0	27734(60.4)	12769(100)	6658	1277(37.7)	2802(87.7)	1109(1.6)	–	3119(28.2)
N1	4503(9.8)	–	–	2107(62.3)	392(12.3)	965(14.0)	–	1039(9.4)
N2	10811(23.6)	–	–	–	–	–	–	5001(45.2)
N3	2856(6.2)	–	–	–	–	–	956(49.6)	1900(17.20)

SD: Standard deviation.

**Table 2:** Mean(SD) of age at diagnosis, for all Surveillance, Epidemiology and End Results(SEER) and in-institute participants, by stage and other clinical characteristics.

Characteristics	All patient N = 45904	Stage IA N = 12769	Stage IB N = 6658	Stage IIA N = 3384	Stage IIB N = 3194	Stage IIIA N = 6911	Stage IIIB N = 1929	Stage IV N = 11059
Race-mean(SD)								
Caucasian	69.59(10.26)	69.75(9.72)	70.69(10.16)	69.36(10.25)	69.63(10.07)	69.36(10.32)	68.54(10.59)	69.12(10.81)
African-American	66.22(10.29)	66.57(9.44)	67.44(10.28)	66.04(10.68)	66.59(10.66)	66.34(10.08)	65.34(10.75)	65.48(10.69)
Asian	66.25(11.28)	64.93(11.16)	66.75(10.55)	66.72(11.04)	67.56(10.71)	65.98(10.80)	67.33(11.92)	67.28(12.29)
Sex-mean(SD)								
Male	69.21(10.19)	69.72(9.53)	70.41(9.94)	68.92(10.19)	69.25(10.02)	68.90(10.31)	68.06(10.69)	68.59(10.69)
Female	68.74(10.65)	68.60(10.23)	69.71(10.62)	68.64(10.70)	69.18(10.44)	68.56(10.54)	68.03(10.83)	68.44(11.31)
Grade-mean(SD)								
Well-differentiated	69.00(10.92)	68.26(10.53)	69.82(11.35)	68.57(12.51)	69.20(10.54)	69.67(11.03)	69.70(10.49)	71.10(11.28)
Moderately-differentiated	69.06(10.24)	69.00(9.7)	70.12(9.96)	68.70(10.36)	69.72(9.98)	68.45(10.37)	67.99(10.81)	68.83(11.03)
Poorly-differentiated	68.91(10.40)	70.31(9.53)	70.10(10.14)	68.94(9.95)	68.80(10.28)	68.81(10.34)	67.99(10.76)	68.06(10.85)
undifferentiated	68.15(10.67)	68.67(9.05)	68.65(12.05)	68.58(8.78)	66.51(11.05)	69.80(10.28)	65.36(8.96)	67.70(11.32)
Histology-mean(SD)								
Squamous cell carcinoma	70.56(9.60)	71.46(8.51)	71.71(9.22)	69.63(9.82)	70.72(9.54)	69.85(10.02)	68.92(10.26)	70.35(9.97)
Adenocarcinoma	68.14(10.75)	68.26(10.27)	69.19(10.72)	68.18(10.80)	68.10(10.56)	67.88(10.63)	67.09(11.19)	67.64(11.32)
Size-mean(SD)								
size ≤ 1 cm	67.37(9.78)	67.44(9.66)	65.04(10.21)	67.03(8.64)	68.00(9.69)	68.54(9.22)	68.04(9.23)	67.25(11.01)
1 cm < size ≤ 2 cm	68.37(9.9)	68.71(9.83)	67.63(9.63)	67.04(9.82)	68.50(9.67)	67.66(9.99)	68.11(10.09)	68.16(10.54)
2 cm < size ≤ 3 cm	69.45(10.38)	70.25(10.12)	69.63(10.34)	67.76(10.15)	69.51(9.54)	68.68(10.28)	69.30(10.78)	68.53(11.19)
3 cm < size ≤ 5 cm	69.50(10.72)	–	70.98(10.29)	67.76(10.78)	70.17(10.04)	69.14(10.54)	67.90(11.18)	68.59(11.11)
5 cm < size ≤ 7 cm	69.31(10.77)	–	–	70.84(10.3)	69.14(10.73)	69.17(10.58)	68.33(10.77)	68.90(11.05)
size > 7 cm	68.30(10.61)	–	–		69.11(10.71)	68.43(10.63)	67.09(10.40)	68.20(10.59)
Site								
Upper lobe	68.79(10.33)	69.14(9.83)	69.79(10.18)	68.81(10.16)	68.51(10.16)	68.51(10.34)	67.91(10.64)	68.21(10.94)
Middle lobe	68.36(10.76)	68.39(10.39)	69.19(10.62)	68.56(10.07)	70.76(10.11)	68.15(10.86)	67.93(11.63)	67.19(11.44)
Lower lobe	69.66(10.42)	69.14(10.05)	70.81(10.28)	68.86(10.81)	70.54(10.12)	69.59(10.31)	69.41(10.61)	69.70(10.88)
Main bronchus	65.82(11.22)	62.18(12.08)	66.36(13.51)	67.44(11.49)	64.66(10.8)	66.02(11.37)	64.63(10.95)	66.10(10.81)
T stage-mean(SD)								
T1a	68.36(9.82)	68.48(9.80)	–	67.08(9.92)	–	67.59(9.61)	67.52(10.77)	68.51(10.13)
T1b	69.74(10.29)	70.20(10.15)	–	68.59(9.76)	–	68.72(10.46)	69.76(11.19)	68.43(11.01)
T2a	69.26(10.58)	–	70.05(10.30)	67.32(10.67)	–	68.24(10.67)	68.90(10.64)	68.76(11.06)
T2b	69.62(10.71)	–	–	70.84(10.26)	68.65(10.82)	68.87(10.69)	70.00(11.03)	68.95(11.05)
T3	68.93(10.56)	–	–	–	69.29(10.13)	68.65(10.57)	67.92(10.67)	68.83(10.93)
T4	68.37(10.76)	–	–	–	–	69.87(9.92)	67.62(10.69)	68.02(11.05)
N stage-mean(SD)								
N0	69.61(10.20)	69.08(9.95)	70.05(10.30)	70.84(10.26)	69.29(10.13)	70.36(9.87)	–	70.33(10.97)
N1	67.86(10.42)	–	–	67.54(10.34)	68.66(10.82)	68.19(10.21)	–	67.89(10.61)
N2	68.31(10.75)	–	–	–	–	68.48(10.54)	67.9(10.56)	68.23(10.99)
N3	67.17(10.83)	–	–	–	–	–	68.19(10.94)	66.65(10.73)

SD: Standard deviation.

**Table 3:** Mean (SD) of age at diagnosis, for all Surveillance, Epidemiology and End Results (SEER) and in-institute participants by ethnicity.

Characteristics	Caucasian n = 37532	African-American n = 4745	Asian n = 3627
Sex-mean(SD)			
Male	69.75(10.04)	66.64(9.99)	67.13(11.08)
Female	69.43(10.46)	65.75(10.58)	65.33(11.42)
Grade-mean(SD)			
Well-differentiated	69.56(10.72)	66.55(11.03)	65.78(11.83)
Moderately-differentiated	69.83(9.99)	66.37(10.20)	65.57(11.10)
Poorly-differentiated	69.41(10.30)	66.04(10.19)	67.64(11.21)
Undifferentiated	68.49(10.94)	64.63(9.26)	68.91(8.13)
Histology-mean(SD)			
Squamous cell carcinoma	70.87(9.52)	68.81(9.58)	68.92(10.31)
Adenocarcinoma	68.87(10.58)	64.8(10.39)	65.51(11.43)
Site			
Upper lobe	69.40(10.16)	66.02(10.26)	66.21(11.17)
Middle lobe	68.95(10.60)	66.26(10.45)	66.31(11.58)
Lower lobe	70.29(10.22)	66.88(10.37)	66.34(11.33)
Main bronchus	66.11(11.31)	63.99(9.53)	65.55(13.4)
T stage-mean(SD)			
T1a	69.05(9.6)	66.37(9.25)	63.76(10.99)
T1b	70.32(10.13)	66.41(10.14)	67.25(11.03)
T2a	69.95(10.42)	66.42(10.71)	66.19(10.72)
T2b	69.99(10.59)	67.15(10.63)	68.78(11.78)
T3	69.47(10.39)	65.97(10.44)	67.34(11.60)
T4	68.95(10.61	65.4(10.41)	66.97(12.01)
N stage-mean(SD)			
N0	70.21(10.05)	66.82(10.12)	66.57(11.18)
N1	68.41(10.33)	65.54(10.32)	65.37(10.66)
N2	68.97(10.59)	65.78(10.50)	65.55(11.55)
N3	67.63(10.78)	64.64(10.37)	67.16(11.55)

SD: Standard deviation.

**Table 4:** Mean(SD) of Tumor size(mm) by patient demographics.

	All patients n = 45904	Caucasian n = 37532	African-American n = 4745	Asian n = 3627
Race-mean(SD)				
Caucasian	35.78(21.36)	–	–	–
African-American	38.89(22.20)	–	–	–
Asian	33.46(19.60)	–	–	–
Sex-mean(SD)				
Male	38.63(22.05)	38.44(22.02)	41.64(22.80)	36.61(20.91)
Female	33.13(20.22)	33.08(20.31)	35.87(21.11)	30.04(17.43)
Grade-mean(SD)				
Well-differentiated	25.71(16.97)	25.74(16.92)	26.62(17.12)	24.62(17.23)
Moderately-differentiated	33.86(20.19)	33.83(20.29)	36.06(21.27)	31.85(17.91)
Poorly-differentiated	41.73(22.05)	41.39(22.06)	44.23(22.39)	41.53(20.85)
Undifferentiated	42.70(23.25)	43.00(23.57)	46.07(24.12)	34.19(15.21)
Histology-mean(SD)				
Squamous cell carcinoma	42.47(22.37)	42.01(22.36)	45.64(22.86)	43.58(20.94)
Adenocarcinoma	32.41(19.91)	32.26(19.92)	35.20(20.93)	30.65(18.24)
Site-mean(SD)				
Upper lobe	35.73(21.41)	35.51(21.36)	38.98(22.50)	33.36(19.79)
Middle lobe	31.87(19.72)	32.25(20.08)	34.67(20.54)	26.67(15.09)
Lower lobe	36.00(21.16)	35.90(21.26)	38.13(21.32)	34.74(19.80)
Main bronchus	49.49(20.66)	49.10(20.62)	53.14(21.62)	46.51(17.52)

SD: Standard deviation.

**Table 5:** Estimates from a multiple linear regression model for age, adjusting for gender, ethnicity (all patients) tumor location, and histology type among patients Stages I,II,III disease.

Age difference(years): Stages I,II,III(T1~3/Nx/M0)	All patients n = 34845	Caucasian n = 28657	African-American n = 3322	Asian n = 2866
Adjusted mean				
age(years),stage IA	68.96(68.93~68.99)	69.59(69.57~69.61)	66.19(66.08~66.30)	65.46(65.38~65.53)
IB vs. IA	0.13(0.09~0.18), p < 0.001	0.13(0.10~0.17), p < 0.001	0.37(0.18~0.56), p < 0.001	0.43(0.31~0.55), p < 0.001
IIA vs. IA	0.25(0.19~0.32), p < 0.001	0.25(0.22~0.30), p < 0.001	0.22(−0.01~0.45), p = 0.062	0.98(0.83~1.14), p < 0.001
IIB vs. IA	0.39(0.33~0.46), p < 0.001	0.34(0.30~0.4), p < 0.001	0.59(0.34~0.83), p < 0.001	1.09(0.91~1.28), p < 0.001
IIIA vs. IA	0.58(0.52~0.65), p < 0.001	0.56(0.50~0.62), p < 0.001	0.54(0.36~0.73), p < 0.001	0.95(0.83~1.08), p < 0.001
IIIB vs. IA	0.83(0.76~0.91), p < 0.001	0.80(0.73~0.88), p < 0.001	1.00(0.74~1.26), p < 0.001	1.38(1.15~1.61), p < 0.001
Adjusted mean				
age(years),T1a	68.92(68.89~68.95)	69.56(69.54~69.57)	66.16(66.02~66.29)	65.40(65.32~65.48)
T1b vs. T1a	0.08(0.03~0.14), p = 0.002	0.08(0.05~0.11), P < 0.001	0.04(−0.17~0.24), p = 0.727	0.49(0.35~0.62), P < 0.001
T2a vs. T1a	0.15(0.10~0.19), P < 0.001	0.18(0.15~0.21), P < 0.001	0.39(0.21~0.58), P < 0.001	0.59(0.48~0.71), P < 0.001
T2b vs. T1a	0.63(0.56~0.70), P < 0.001	0.52(0.48~0.57), P < 0.001	1.25(0.99~1.52), P < 0.001	1.42(1.23~1.6), P < 0.001
T3 vs. T1a	0.62(0.57~0.69), P < 0.001	0.35(0.31~0.38), P < 0.001	0.76(0.54~0.97), P < 0.001	1.23(1.08~1.38), P < 0.001
T4 vs. T1a	0.92(0.85~0.99), P < 0.001	0.84(0.77~0.91), P < 0.001	0.92(0.67~1.17), P < 0.001	1.21(1.02~1.41), P < 0.001
Adjusted mean				
age(years),N0	69.09(69.07~69.11)	69.70(69.69~69.71)	66.45(66.36~66.53)	65.76(65.7~65.82)
N1 vs. N0	0.09(0.03~0.15), p = 0.002	0.12(0.08~0.16), p < 0.001	−0.03(−0.2~0.25), p = 0.826	0.73(0.58~0.89), p < 0.001
N2 vs. N0	0.12(0.08~0.17), p < 0.001	0.16(0.12~0.19), p < 0.001	0.25(0.08~0.43), p = 0.004	0.69(0.57~0.82), p < 0.001
N3 vs. N0	0.56(0.45~0.66), p < 0.001	0.43(0.1~0.24), p < 0.001	0.77(0.43~1.1), p < 0.001	0.93(0.6~1.25), p < 0.001

Values given are mean differences in ages by tumor size and stage of disease, with 95%CIs.

**Table 6:** Estimates from a multiple linear regression model for age, adjusting for gender, race (all patients) tumor location, histology type among patients Stages I, II, III disease.

Age difference (years):Stages I,II,III (Tx/Nx/M0)	Adenocarcinoma n = 12381	Squamous cell carcinoma n = 22464
Adjusted mean		
age(years),stage IA	68.25 (68.21~68.29)	71.46(71.42~71.50)
IB vs. IA	0.91(0.53~1.28), p < 0.001	0.24(−0.22~0.71), p = 0.307
IIA vs. IA	−0.06(−0.56~0.44), p = 0.813	0.75(0.21~1.15), p = 0.008
IIB vs. IA	−0.16(−0.68~0.35), p = 0.524	1.82(1.26~2.46), p < 0.001
IIIA vs. IA	0.38(−0.76~0.1), p = 0.057	1.64(1.23~2.12), p < 0.001
IIIB vs. IA	1.17(0.47~1.87), p = 0.001	2.52(1.90~3.10), p < 0.001
Adjusted mean		
age(years),T1a	67.57(67.53~67.61)	70.96(70.94~71.01)
T1b vs. T1a	0.17(−0.28~0.63), p = 0.453	0.45(−0.19~1.10), p = 0.109
T2a vs. T1a	1.04(0.68~1.39), p = 0.036	−0.21(−0.76~0.27), p = 0.391
T2b vs. T1a	1.44(0.8~2.08), P < 0.001	−0.39(−1.00~0.21), p = 0.201
T3 vs. T1a	1.66(1.26~2.05), p < 0.001	0.57(0.32~1.12), p = 0.038
T4 vs. T1a	0.92(0.31~1.5), p = 0.003	1.82(1.21~2.46), p < 0.001
Adjusted mean		
age(years),N0	68.60(68.54~68.66)	71.43(71.39~71.46)
N1 vs. N0	1.14(0.67~1.62), p < 0.001	1.89(1.03~2.76), p < 0.001
N2 vs. N0	1.08(0.69~1.48), p < 0.001	1.92(1.54~2.46), p < 0.001
N3 vs. N0	1.62(0.7~2.56), p = 0.001	3.01(2.49~3.53), p < 0.001

Values given are mean differences in ages by tumor size and stage of disease, with 95%CIs.

**Table 7:** Estimates from a multiple linear regression model for age, adjusting for gender, ethnicity (all patients), grade, tumor location and histology type among patients Stages I,II,III disease.

Age difference(years): Stages I,II,III	All patients n = 34845	Caucasian n = 28657	African-American n = 3322	Asian n = 2866
Adjusted mean age(years)				
tumor ≤ 1 cm	68.83(68.77~68.9)	69.52(69.48~69.55)	66.04(65.76~66.32)	65.17(65.03~65.31)
1~2 cm vs. ≤ 1 cm	0.06(−0.01~0.13), p = 0.096	0.02(−0.01~0.07),P = 0.256	−0.005(−0.31~0.30),p = 0.974	0.37(0.2~0.53), p < 0.001
2~3 cm vs. ≤ 1 cm	0.13(0.05~0.21), p = 0.001	0.1(0.06~0.15), p < 0.001	0.18(−0.15~0.52), p = 0.282	0.69(0.52~0.87), p < 0.001
3~4 cm vs. ≤ 1 cm	0.34(0.26~0.43), p < 0.001	0.29(0.24~0.35), p < 0.001	0.67(0.30~1.04), p < 0.001	1.13(0.94~1.32), p < 0.001
4~5 cm vs. ≤ 1 cm	0.59(0.49~0.68), p < 0.001	0.47(0.41~0.53), p < 0.001	0.85(0.47~1.25), p < 0.001	1.28(1.06~1.51), p < 0.001
5~6 cm vs. ≤ 1 cm	0.73(0.64~0.83), p < 0.001	0.56(0.50~0.62), p < 0.001	1.17(0.75~1.60), p < 0.001	1.69(1.41~1.9), p < 0.001
6~7 cm vs. ≤ 1 cm	0.85(0.72~0.91), p < 0.001	0.68(0.61~0.75), p < 0.001	1.24(0.81~1.67), p < 0.001	1.76(1.63~2.17), p < 0.001
7~8 cm vs. ≤ 1 cm	0.76(0.64~0.88), p < 0.001	0.70(0.64~0.75), p < 0.001	1.49(1.03~1.95), p < 0.001	1.81(1.49~2.11), p < 0.001
>8 cm vs. ≤ 1 cm	0.85(0.64~0.89), p < 0.001	0.79(0.71~0.85), p < 0.001	1.40(0.94~1.86), p < 0.001	1.79(1.41~2.16), p < 0.001

Values given are mean differences in ages by tumor size and stage of disease, with 95%CIs.

**Table 8:** Characteristic of small invasive tumor in SEER database and our institute.

Stage-Number (%)	0~3 mmn = 89	3~5 mmn = 191	5~7 mmn = 361	≤3 cmn = 23552
T2a	1(1.1)	12(6.3)	29(8.0)	–
T3	11(12.4)	20(10.4)	22(6.0)	2238(9.5)
T4	4(4.4)	8(4.1)	12(3.3)	1465(6.2)
N1	5(5.6)	7(3.7)	8(2.3)	1739(7.4)
N2	10(1.1)	6(3.1)	24(6.6)	3154(13.4)
N3	–	4(2.0)	3(0.8)	733(3.1)
