# Supplementary material for: Time-to-Progression of NSCLC from Early to Advanced Stages: An Analysis of data from SEER Registry and a Single Institute
Source: Sci Rep. 2016 Jun 27;6:28477. doi: 10.1038/srep28477 (PMC4921917; doi:10.1038/srep28477)
Supplement: Supplementary Information [file srep28477-s1.doc]

Supplementary Tables for

**Time-to-Progression of NSCLC from Early to Advanced Stages: An Analysis of data from SEER Registry and a Single Institute**

**Author list:** Ping Yuan^1^, Ph.D, Jin Lin Cao^1^, Ph.D, Azmat Rustam MD, Chong Zhang^1^, MD, Xiao Shuai Yuan^1^, Ph.D, Fei Chao Bao^1^, MD, Wang Lv^1^, M.D., Jian Hu^1^. MD.,Ph.D

^1^ Department of Thoracic surgery, The First Affiliated Hospital, School of Medicine, Zhejiang University, Hangzhou, China, 310003

**Corresponding author:**

Jian Hu,

Department of Thoracic surgery, The First Affiliated Hospital, School of Medicine, Zhejiang University, Hangzhou, China, 310003

Fax: 86057186995818,

E-mail: hujian_med2@163.com

| **Supplementary Table 1. Searching code in Surveillance, Epidemiology and End Results (SEER) participants** | |
| --- | --- |
| Site and Morphology.Site recode ICD-O-3/WHO 2008: | Lung and Bronchus |
| Race and Age (case data only).Age at diagnosis: | 30-120 |
| Site and Morphology.Grade: | Well differentiated; Grade I Moderately differentiated; Grade II Poorly differentiated; Grade III Undifferentiated; anaplastic; Grade IV |
| Site and Morphology.Primary Site - labeled: | C34.0-Main bronchus C34.1-Upper lobe, lung C34.2-Middle lobe, lung C34.3-Lower lobe, lung |
| Site and Morphology.Histology recode - broad groupings: | 8050-8089: squamous cell neoplams 8140-8389: adenomas and adenocarcinomas |
| Site and Morphology.Diagnostic Confirmation | Positive histology Positive exfoliative cytology, no positive histology Positive microscopic confirm, method not specified |
| Stage - AJCC.Derived AJCC Stage Group, 7th ed (2010+): | I','IA','IB','II','IIA','IIB','IIIA','IIIB','IV' |
| Stage - TNM.Derived AJCC T, 7th ed (2010+): | 'T1a','T1b','T2a','T2b','T3','T4' |
| Stage - TNM.Derived AJCC N, 7th ed (2010+): | 'N0','N1','N2','N3' |
| Stage - TNM.Derived AJCC M, 7th ed (2010+): | 'M0','M1' |
| Race and Age (case data only).Race/ethnicity: | White','Black','Chinese','Japanese','Filipino','Korean (1988+)','Vietnamese (1988+)','Laotian (1988+)','Hmong (1988+)','Kampuchean (1988+)','Thai (1994+)','Asian Indian (2010+)','Pakistani (2010+)','Other Asian (1991+)' |

| **Supplementary Table 2: TNM staging of NSCLC 7th edition and stage grouping** | |
| --- | --- |
| Primary tumor (T) | |
| T1a: | tumor ≤ 2 cm in diameter. |
| T1b: | tumor > 2 cm but ≤ 3 cm in diameter. |
| T2a: | tumor > 3 cm but ≤ 5 cm in diameter. |
| T2b: | tumor > 5 cm but ≤ 7 cm in diameter. |
| T3: | tumor >7 cm in diameter, or tumor with any of the following features: |
|  | Direct invasion of the chest wall, diaphragm, phrenic nerve. |
|  | Direct invasion of mediastinal pleura or parietal pericardium. |
|  | Associated atelectasis or obstructive pneumonitis of the entire lung. |
|  | tumor within the main bronchus <2 cm to the carina, without involvement of the carina |
|  | Satellite tumor nodules in the same lobe. |
| T4: | tumor of any size that has any of the following features: |
|  | Invasion of the mediastinum. |
|  | Invasion the heart or great vessels. |
|  | Invasion of the trachea, oesophagus or recurrent laryngeal nerve. |
|  | Invasion of a vertebral body or carina. |
|  | Separate tumor nodules in a different ipsilateral lobe. |
| Regional lymph nodes (N) | |
| N0: | No regional lymph node metastasis. |
| N1: | Involvement of ipsilateral hilar or peri-bronchial nodes. |
| N2: | Involvement of ipsilateral mediastinal or subcarinal nodes. |
| N3: | Involvement of contralateral mediastinal or hilar nodes, OR involvement of ipsilateral/contralateral  scalene or supraclavicular nodes. |
| Distant Metastasis (M) | |
| M0: | No distant metastasis. |
| M1: | Distant metastasis present. |
| Stage Grouping | |
| IA: | T1a/T1b, N0M0 |
| IB: | T2aN0M0 |
| IIA: | T1a/T1b, N1M0, T2aN1M0, T2bN0M0 |
| IIB: | T2bN1M0, T3N0M0 |
| IIIA: | T1/T2, N2M0, T3, N1/N2, M0, T4, N0/N1, M0 |
| IIIB: | T4N2M0, Any T, N3, M0 |
| IV: | Any T, Any N, M1 |

| **Supplementary Table 3. Comparison of tumor characteristics in Asian patients in SEER database and our institute** | | | |
| --- | --- | --- | --- |
|  | SEER database n = 2547 | In-institute n = 1080 | P value |
| **Age at diagnosis-mean(SD)** | 68.60(11.16) | 60.71(9.5) | <0.001 |
| **Sex-mean** |  |  | 0.113 |
| Male | 1304(51.2) | 584(54.1) |  |
| Female | 1243(48.8) | 496(45.9) |  |
| **Grade** |  |  | <0.001 |
| Well-differentiated | 511(20.1) | 134(12.4) |  |
| Moderately-differentiated | 1066(41.9) | 807(74.7) |  |
| Poorly-differentiated | 940(36.9) | 137(12.7) |  |
| Undifferentiated | 30(1.2) | 2(0.2) |  |
| **Histology-mean** |  |  | <0.001 |
| Squamous cell carcinoma | 509(20.0) | 279(25.8) |  |
| Adenocarcinoma | 2038(80.0) | 801(74.2) |  |
| **Site-mean** |  |  | <0.001 |
| Upper lobe | 1489(58.5) | 598(55.4) |  |
| Middle lobe | 188(7.4) | 98(9.1) |  |
| Lower lobe | 825(32.4) | 382(35.4) |  |
| Main bronchus | 45(1.8) | 2(0.2) |  |
| **SD: Standard deviation** |  |  |  |

| **Supplementary Table 4. Estimates from a multiple linear regression model for age, adjusting for patient ethnicity (all patients), sex, tumor location, grade and histology type among patients with stage I to IV disease.** | | | | |
| --- | --- | --- | --- | --- |
| **Age difference (years):** | **All patients n=45904** | **Caucasian n=37532** | **African-American n=4745** | **Asian n=3627** |
| **Adjusted mean  age (years), stage IA** | 68.93 (68.90~68.95) | 69.56 (69.54~69.57) | 66.05 (65.95~66.16) | 65.64 (65.56~65.72) |
| IB vs. IA | 0.02 (0.03~0.13), p=0.001 | 0.08 (0.05~0.11), p<0.001 | 0.30 (0.12~0.48), p=0.001 | 0.44 (0.3~0.58), p<0.001 |
| IIA vs. IA | 0.17 (0.11~0.23), P<0.001 | 0.16 (0.12~0.21), p<0.001 | 0.09 (-0.13~0.32), p=0.394 | 1.11 (0.93~1.3), p<0.001 |
| IIB vs. IA | 0.33 (0.26~0.39), P<0.001 | 0.18 (0.14~0.22), p<0.001 | 0.48 (0.24~0.72), p<0.001 | 1.29 (1.06~1.5), p<0.001 |
| IIIA vs. IA | 0.39 (0.33 to 0.46), p<0.001 | 0.15 (0.12~0.18), p<0.001 | 0.38 (0.20~0.56), p<0.001 | 1.01 (0.86~1.16), p<0.001 |
| IIIB vs. IA | 0.26 (0.22 to 0.31), p<0.001 | 0.26 (0.2~0.31), p<0.001 | 0.84 (0.58~1.09), p<0.001 | 1.48 (1.22~1.74), p<0.001 |
| IV vs. IA | -0.07 (-0.2~0.06), p=0.296 | -0.06 (-0.15~0.04), p=0.233 | -0.07 (-0.22~0.08), p=0.379 | 0.79 (0.07~0.92), p <0.001 |
| Values given are mean differences in ages by tumor size and stage of disease, with 95% CIs. | | | | |

| **Supplementary Table 5. Estimates from a multiple linear regression model for age, adjusting for gender, race, tumor location, histology type among Asian patients in SEER database and our institute with Stages I, II, III disease.** | | |
| --- | --- | --- |
| Age difference (years): Stages I, II,III (Tx/Nx/M0) | SEER database n=1801 | In-institute n=1065 |
| **Adjusted mean age(years),stage IA** | 68.63(68.62~68.65) | 60.11(60.08~60.14) |
| IB vs. IA | 0.31(0.13~0.48),p=0.001 | 0.49(0.27~0.72),p<0.001 |
| IIA vs. IA | 0.79(0.56~1.03),p<0.001 | 1.14(0.87~1.52),p<0.001 |
| IIB vs. IA | 0.89(0.63~1.14),p<0.001 | 1.34(1.02~1.79),p<0.001 |
| IIIA vs. IA | 0.92(0.64~1.21),p<0.001 | 1.11(0.88~1.34),p<0.001 |
| IIIB vs. IA | 1.46(1.18~1.74),p<0.001 | 0.57(-0.27~1.43),p=0.186 |
| **Adjusted mean age(years),T1a** | 68.66(68.63~68.73) | 60.12(60.09~60.15) |
| T1b vs. T1a | 0.08(-0.10~0.27),p=0.37 | 0.99(0.73~1.24),p<0.001 |
| T2a vs. T1a | 0.34(0.16~0.52),p<0.001 | 0.74(0.54~0.94),p<0.001 |
| T2b vs. T1a | 1.41(1.13~1.69),p<0.001 | 1.56(1.36~2.27),p<0.001 |
| T3 vs. T1a | 1.02 (0.78~1.25),p<0.001 | 1.64(1.37~2.13),p<0.001 |
| T4 vs. T1a | 1.16(0.88~1.43),p<0.001 | 1.41(0.89~1.92),p<0.001 |
| **Adjusted mean age(years),N0** | 68.90(68.87~68.93) | 60.49(60.46~60.52) |
| N1 vs. N0 | 0.45(0.21~0.70),p<0.001 | 0.89(0.62~1.16),p<0.001 |
| N2 vs. N0 | 0.69(0.49~0.88),p<0.001 | 0.62(0.38~0.85),p<0.001 |
| N3 vs. N0 | 0.95(0.56~1.33),p<0.001 | -- |
| **Values given are mean differences in ages by tumor size and stage of disease, with 95% CIs.** | | |

| **Supplementary Table 6. Estimates from three multiple linear regression models for age, adjusting for gender, ethnicity(all patients) tumor location, histology type among patients with stage I, stage II, stage III disease respectively.** | | | | |
| --- | --- | --- | --- | --- |
| Age difference(years): | All patients n=34845 | Caucasian n=28657 | African-American n=3322 | Asian n=2866 |
| **Adjusted mean age(years) in stage I(T1a)** | 69.24(69.2~69.29) | 69.92(68.89~69.95) | 66.67(66.52~66.83) | 65.12(64.99~65.24) |
| I(T1b) vs. I(T1a) | 0.22( 0.14~0.29),p<0.001 | 0.18(0.13~0.22),p<0.001 | 0.14(-0.12~0.40),p=0.299 | 0.6(0.38~0.83),p<0.001 |
| I(T2a) vs. I(T1a) | 0.34(0.28~0.40),p<0.001 | 0.21(0.29~0.37),p<0.001 | 0.52(0.28~0.75),p<0.001 | 0.88(0.68~1.07),p<0.001 |
| **Adjusted mean age(years) in stage II(T1a**) | 68.75(68.62~68.87) | 69.22(69.12~69.31) | 65.22(64.56~65.87) | 67.17(66.61~67.72) |
| II(T1b) vs. II(T1a) | -0.001(-0.18~0.18),p=0.995 | 0.08(-0.05~0.21),p=0.221 | -0.53(-1.37~0.31),p=0.211 | -0.13(-0.77~0.51),p=0.68 |
| II(T2a) vs. II(T1a) | -0.003(-0.17~0.17),p=0.975 | 0.21(0.09~0.33),p<0.001 | 0.70(-0.15~1.54),p=0.106 | -0.33(-0.77~0.1),p=0.134 |
| II(T2b) vs. II(T1a) | 0.52(0.37~0.67),p<0.001 | 0.56(0.45~0.68),p<0.001 | 1.94(1.08~2.8),p<0.001 | -0.09(0.58~0.38),p=0.688 |
| II(T3) vs. II(T1a) | 0.49(0.35~0.63),p<0.001 | 0.44(0.34~0.55),p<0.001 | 1.19(0.39~2.00),p=0.004 | 0.02(-0.4~0.43),p=0.941 |
| **Adjusted mean age(years) in stage III(T1a**) | 68.35(68.25~68.45) | 68.89(68.83~68.96) | 65.32(64.94~65.71) | 66.10(65.83~66.38) |
| III(T1b) vs. III(T1a) | -0.22(-0.37~0.07),p=0.004 | -0.02(-0.11~0.07),p=0.720 | -0.23(-0.3~0.77),p=0.388 | -0.05(-0.35~0.25),p=0.75 |
| III(T2a) vs. III(T1a) | 0.05(-0.08~0.18),p=0.466 | 0.18(0.09~0.26),p<0.001 | 0.52(0.04~1.00),p=0.032 | -0.04(-0.31~0.23),p=0.758 |
| III(T2b) vs. III(T1a) | 0.40(0.26~0.54),p<0.001 | 0.40(0.31~0.49),p<0.001 | 1.28(0.74~1.83),p<0.001 | 0.27(-0.077~0.61),p=0.121 |
| III(T3) vs. III(T1a) | 0.42(0.30~0.54),p<0.001 | 0.40(0.33~0.49),p<0.001 | 0.99(0.52~1.45),p<0.001 | 0.25(-0.05~0.55),p=0.104 |
| III(T4) vs. III(T1a) | 0.72(0.60~0.84),p<0.001 | 0.69(0.62~0.78),p<0.001 | 0.89(0.41~1.37), p<0.001 | 0.19(-0.13~0.51),p=0.253 |
| **Values given are mean differences in ages by tumor size and stage of disease, with 95%CIs.** | | | | |

| **Supplementary Table 7. Estimates from a multiple linear regression model for age, adjusting for gender, ethnicity (all patients), grade, tumor location and histology type throughout Stages I,II,III disease.** | | | | |
| --- | --- | --- | --- | --- |
| **Age difference(years): Stages I,II,III** | **All patients n=34845** | **Caucasian n=28657** | **African-American n=3322** | **Asian n=2866** |
| **Adjusted mean age(years) stage I(T1a)** | 68.91(68.88~68.94) | 69.48(69.4~69.55) | 66.16(66.02~66.30) | 65.33(65.25~65.41) |
| II(T1b) vs. I(T1a) | 0.11(-0.04~0.25),p=0.167 | 0.07(-0.01~0.16),p=0.084 | -0.08(-0.21~0.05),p=0.212 | 0.52(0.13~0.92),p=0.01 |
| II(T2a) vs. I(T1a) | 0.08(-0.02~0.18),p=0.106 | 0.21(0.15~0.27),p<0.001 | 0.09(-0.26~0.44),p=0.608 | 0.88(0.68~1.08),p<0.001 |
| II(T2b) vs. I(T1a) | 0.67(0.58~0.75),p<0.001 | 0.53(0.48~0.59),p<0.001 | 1.15(0.83~1.47),p<0.001 | 1.49(1.27~1.7),p<0.001 |
| II(T3) vs. I(T1a) | 0.4(0.33~0.47),p<0.001 | 0.26(0.22~0.30),p<0.001 | 0.5(0.23~0.77),p<0.001 | 1.16(0.97~1.35),p<0.001 |
| III(T1b) vs. I(T1a) | -0.21(-0.34~0.09),p=0.001 | -0.06(-0.13~0.009),p=0.088 | -0.04(-0.37~0.45),p=0.859 | 0.93(0.68~1.18),p<0.001 |
| III(T2a) vs. I(T1a) | 0.11(0.03~0.19),p=0.006 | 0.18(0.12~0.22),p<0.001 | 0.38(0.08~0.67),p=0.011 | 0.81(0.63~0.99),p<0.001 |
| III(T2b) vs. I(T1a) | 0.58(0.47~0.70),p<0.001 | 0.49(0.42~0.56),p<0.001 | 1.3(0.91~1.70),p<0.001 | 1.47(1.19~1.76),p<0.001 |
| III(T3) vs. I(T1a) | 0.50(0.42~0.58),p<0.001 | 0.45(0.40~0.50),p<0.001 | 0.92(0.65~1.19),p<0.001 | 1.44(1.26~1.63),p<0.001 |
| III(T4) vs. I(T1a) | 0.79(0.7~0.88),p<0.001 | 0.7(0.66~0.75),p<0.001 | 0.88(0.62~1.13),p<0.001 | 1.28(1.09~1.48),p<0.001 |
| **Adjusted mean age(years) stage II(T1a)** | 69.00(68.87~69.15) | 69.53(69.44~69.63) | 65.71(65.20~65.23) | 66.13(65.63~66.64) |
| III(T1b) vs. II(T1a) | -0.31(-0.50~0.11),p=0.003 | -0.04(-0.16~0.08),p=0.53 | 0.48(-0.22~1.19),p=0.177 | 0.12(-0.42~0.68),p=0.649 |
| III(T2a) vs. II(T1a) | 0.02(-0.16~0.21),p=0.807 | 0.2(0.09~0.32),p=0.001 | 0.82(0.16~1.48),p=0.015 | 0.009(-0.53~0.55),p=0.972 |
| III(T2b) vs. II(T1a) | 0.49(0.3~0.68),p<0.001 | 0.51(0.38~0.64),p<0.001 | 1.74(1.01~2.48),p<0.001 | 0.68(0.04~1.31),p=0.037 |
| III(T3) vs. II(T1a) | 0.41(0.23~0.59),p<0.001 | 0.48(0.36~0.59),p<0.001 | 1.36(0.72~2.02),p<0.001 | 0.64(0.04~1.24),p=0.036 |
| III(T4) vs. I(T1a) | 0.69(0.53~0.86),p<0.001 | 0.65(0.53~0.76),p<0.001 | 1.32(0.67~1.98),p<0.001 | 0.48(-0.15~1.12),p=0.139 |
| Values given are mean differences in ages by tumor size and stage of disease, with 95%CIs. | | | | |
